# Comparative Transcriptome Analysis Provides Insights into the Molecular Mechanism Underlying the Effect of MeJA Treatment on the Biosynthesis of Saikosaponins in *Bupleurum chinense* DC.

**DOI:** 10.3390/life13020563

**Published:** 2023-02-17

**Authors:** Yanping Mao, Yuping Yang, Yuchan Li, Yiguan Zhang, Ping Wei, Hua Chen, Dabin Hou

**Affiliations:** 1 School of Life Science and Engineering, Southwest University of Science and Technology, Mianyang 621010, China; 2 College of Life Science and Biotechnology, Mianyang Teachers’ College, Mianyang 621000, China; 3 Ecological Security and Protection Key Laboratory of Sichuan Province, Mianyang Teachers’ College, Mianyang 621000, China; 4 Sichuan Institute for Translational Chinese Medicine, Chengdu 610041, China

**Keywords:** comparative transcriptome, MeJA, *Bupleurum chinense* DC., saikosaponin biosynthesis, differentially expressed gene

## Abstract

*Bupleurum chinense* DC. is a well-known traditional Chinese medicinal plant that produces saikosaponins (SSs), which possess hepatoprotective, antipyretic, and anti-inflammatory activities. Methyl jasmonate (MeJA) is a signalling phytohormone that can increase the accumulation of SSs in the root of *Bupleurum* plants. However, the molecular understanding of MeJA-mediated SS biosynthesis is not clear. Therefore, it is necessary to explore the molecular mechanism underlying the response of *B. chinense* DC. to MeJA in roots. In this study, we performed comparative transcriptome analysis of *B. chinense* DC. roots with different MeJA treatment times. In total, 104,057 unigenes were identified, of which 4053 were differentially expressed genes (DEGs). Most of the DEGs were downregulated after MeJA treatment, and GO enrichment analysis showed that they were mainly related to biological processes involved in stress responses and development. A total of 88 DEGs encoding enzymes known to be involved in the SS synthesis pathway were found, and most were significantly downregulated within 24 h. Based on the DEGs, 99 transcription factors (TFs) belonging to the AP2/ERF, WRKY, bZIP, ZFP, and bHLH families with different expression patterns were also identified. Further integrated analysis indicated that 20 DEGs involved in the SS synthesis pathway and 12 DEGs encoding TFs presented strong correlations with the SS contents, and these DEGs may be critical for the biosynthesis and regulation of SSs. These findings will be critical for further study of the response of *B. chinense* DC. to MeJA for SS biosynthesis.

## 1. Introduction

*Bupleurum chinense* DC. is a perennial plant that is widely cultivated in Asia. *B. chinense* DC. and *Bupleurum scorzonerifolium* Willd. are standard herbs used to prepare *Bupleuri Radix* (BR) [1], which is the dried root of plants of the genus *Bupleurum*. BR, as an important herbal material for the treatment of fever, pain, inflammation, influenza, hepatitis, malaria, and menstrual disorders in Asian countries, has been used for more than 2000 years [2]. BR possesses anti-inflammatory, anticancer, antiviral, antipyretic, and other pharmacological effects [3]. The Chinese medicine formula called Qing Fei Pai Du Tang, prepared from *B*. *chinense*, was even able to prevent the progression of mild cases of COVID-19 [4]. The primary active components of BR are SSs (saikosaponins) that are specific to the genus *Bupleurum*. More than 80 saponins have been isolated from BR [5], and SSa (saikosaponin a, Supplementary Figure S1) and SSd (saikosaponin d, Supplementary Figure S1) are regarded as markers for quality evaluation. SS biosynthesis depends on the mevalonate (MVA) pathway and the methylerythritol phosphate (MEP) pathway. 2,3-Oxidosqualene is cyclized to β-amyrin by β-amyrin synthase (β-AS), and then β-amyrin is modified by cytochrome P450 enzymes (P450s) and uridine diphosphate glycosyltransferases (UGTs) for the formation of diverse SSs. Several enzymes involved in SS biosynthesis have been identified, including HMG-CoA reductase (HMGR), squalene epoxidase (SE), β-AS, P450, and UGT [6]. However, the biosynthesis and regulatory mechanisms of SSs remain unclear.

MeJA is a plant stress hormone and can increase resistance in plants by controlling growth and development. An exogenous supply of MeJA can improve plant resistance to salinity stress, drought stress, heavy metal toxicity, chilling stress, and other biotic and abiotic stresses [7]. Additionally, MeJA can regulate gene transcription and the levels of secondary metabolites [8]. MeJA treatment of the leaves of *Chrysanthemum indicum* var. *aromaticum* (*Ci. aromaticum*) change the levels of volatile terpenoids, and the expression of terpene synthetase genes was substantially changed. Exogenous MeJA effectively increased the accumulation of chlorogenic acid and its derivatives in *Gardenia jasminoides* Ellis cells, and 19 DEGs involved in caffeoylquinic acid biosynthesis were identified [9]. These findings provide new ideas for identifying candidate genes and improving metabolite production via external MeJA application and provide the basis for further understanding of the underlying biosynthesis and regulatory mechanisms of these secondary metabolites.

In recent years, with the development of RNA sequencing (RNA-seq), transcriptome analysis has been widely applied to investigate the biosynthesis pathway and candidate genes of secondary metabolites. Numerous metabolic pathways of secondary metabolites in medicinal plants have been studied through transcriptome analysis, and several key genes have been identified, including those in the *Polygonum cuspidatum* [10], *Panax ginseng* [11], *Gastrodiaelata* [12], and *Swertia japonica* [13]. In the genus *Bupleurum*, some studies have been performed based on RNA-seq transcriptome analysis, including comparative analysis between *B. chinense* DC. and *B. scorzonerifolium* Willd. [14,15,16], continuous inflorescence removal treatment in *B. chinense* DC. [17], exogenous abscisic acid and drought stress treatment in *B. chinense* DC. [18,19,20], and integrated analysis of the metabolome and transcriptome in *B. chinense* DC. [21,22]. However, the results remain limited, and there is great research value to further explore the biosynthesis and regulation of SSs in *B. chinense* DC. using RNA-seq.

Previous reports showed that the SS levels were changed after MeJA treatment [23,24], and the expression of some TFs and key genes involved in SS synthesis changed significantly [25,26,27]. Nevertheless, the mechanism underlying the regulation of SS synthesis under MeJA treatment at the transcriptional level remains unknown. Therefore, it is necessary to conduct further research on this topic, gain a better understanding of global gene expression changes in response to MeJA in *B. Chinense* DC., and further explore the key genes related to SS biosynthesis. In this work, we first applied the RNA-seq technique to explore the transcriptional changes that occur in response to MeJA treatment in *B. chinense* DC. Comparative transcriptome analysis of *B. chinense* DC. with different MeJA treatment times was conducted to explore the candidate genes that participate in or affect the synthesis, regulation, and accumulation of SSs at the transcriptional level. This study enhances the understanding of the molecular mechanism of SS biosynthesis, and the findings provide a molecular basis for understanding the mechanism underlying the MeJA response in the *Bupleurum* genus, which will be critical for future research on metabolic engineering and germplasm optimization.

## 2. Materials and Methods

### 2.1. Plant Material and MeJA Treatment

Seeds of *B. chinense* DC. plants of the CBC1 genotype were sown in pots containing a mixture of peat soil (15.5 cm × 14 cm) with four plants per pot and placed in a greenhouse in Mianyang, Sichuan Province, China. A photoperiod of 12 h light and 12 h dark was used, and the ambient temperature was 20 to 24 °C. Three months later, the plants that exhibited consistent growth were selected and treated, and untreated plants were used as the control group (CK). 5 mL of 100 μM MeJA was added to the soil around the root for each plant, and the root samples were collected after treatment at 6 h, 12 h, 24 h, 48 h, and 72 h. Then, all the treated and untreated root samples were washed clean with ice-cold distilled water, and the excess water was blotted dry with sterilized filter paper. For transcriptome sequencing, the samples were flash-frozen in liquid nitrogen and stored at −80 °C. For SS extraction, the samples were dried at 80 °C to constant weight and individually crushed to a fine powder for further use.

### 2.2. RNA Isolation, Library Construction, and Transcriptome Sequencing

Total RNA was extracted from each sample by TRIzol reagent (Invitrogen), and RNA quantification and qualification were performed using a NanoDrop 2000 and the Agilent 2100/4200 system. The mRNA was purified from total RNA using polyT and cleaved into short fragments of 300–350 bp, followed by purification and connection using sequencing adaptors. The template was enriched by PCR, and the PCR product was purified to obtain the final library. After library preparation, the samples were subjected to Illumina sequencing using PE150 (paired-end 150 nt) sequencing.

### 2.3. Transcriptome Assembly and Annotation

The clean reads were obtained from raw reads using in-house Perl scripts by removing reads containing adapters, reads with more than 3 Ns, and low-quality reads (more than 20% nucleotides with Qphred ≤ 5). The clean reads from all the samples were used for de novo transcriptome assembly by Trinity [28]. Unigenes were obtained after assembly, and then functional annotation was performed based on the Gene Ontology (GO), Kyoto Encyclopedia of Genes and Genomes (KEGG), euKaryotic Orthologous Groups (KOG), NCBI nonredundant protein sequences (NR), NCBI nonredundant nucleotide sequence (NT), manually annotated and reviewed protein sequence database (Swiss-Prot), and protein family database (Pfam).

### 2.4. Differential Expression Analysis and Functional Enrichment

Gene expression levels were estimated from the FPKM values, which were calculated using RSEM. Edge R was used for differential expression analysis, and genes with |log2 (fold change)| > 1 and q value < 0.05 were considered DEGs. Then, GO and KEGG enrichment analyses of the DEGs were performed using the GOseq R and KOBAS 3.0 packages and an adjusted p value below 0.05. Heatmaps were produced using the Heatmap package in R. The TFs were predicted using iTAK 1.7 software. The Pearson correlation coefficient was used to evaluate the relationship between the SS content and gene expression, and interaction network visualization was carried out with Cytoscape (version 3.9.1).

### 2.5. Simple Sequence Repeats (SSRs) and Variant Detection

SSRs in the transcriptome were detected using MISA (http://pgrc.ipkgatersleben.de/misa/misa.html (accessed on 10 October 2021)) with default parameters, corresponding to each unit size (unit size/minimum repeat time) set to 1/10, 2/6, 3/5, 4/5, 5/5, and 6/5. Here, 1/10 indicates ten repeat units for mononucleotides, 2/6 indicates six repeat units for dinucleotides, and so on. Samtools software was used for detection of the SNPs and InDels.

### 2.6. Quantitative Real-Time PCR (qRT–PCR) Analysis

The total RNA isolated for RNA-seq analysis was also used for qRT–PCR, and cDNA was synthesized using cDNA Synthesis SuperMix (TransScript, Beijing, China). The specific primers for 12 genes were designed by Primer 5.0 and used for qRT–PCR, which was performed using PerfectStart Green qPCR SuperMix (TransScript) on a CFX96 Real-Time PCR System (Bio-Rad, Hercules, CA, USA). The qRT–PCR conditions were 94 °C for 30 s, followed by 40 cycles of 94 °C for 5 s and 60 °C for 30 s. The expression level was quantified from the cycle threshold (CT) values, and the relative gene expression level was calculated by the 2^−ΔΔCt^ method. All primers used are listed in Supplementary Table S1.

### 2.7. HPLC Analysis

The powder of the dried roots (0.25 g) was dissolved in 25 mL of methanol ammonia solution (95:5, *v/v*), and the solution was then sonicated at 30 °C for 40 min. After cooling to room temperature, the suspension was filtered, and then the filtrate was dried in a water bath at 55 °C. The residue was redissolved in 10 mL of methanol and filtered through a 0.45 µm filter. A 10 µL aliquot of each sample solution was used for injection. The levels of SSa and SSd were determined using a Waters e2695 separation modules system carrying a 2489 UV/V detector with a Waters Symmetry C18 column (250 mm × 4.6 mm, 5 µm). The standards of SSa and SSd for HPLC analysis were purchased from the National Institutes for Food and Drug Control, Beijing, China. The methods and conditions were used as previously described [24].

### 2.8. Statistical Analysis

All the data in this work are presented as the means ± standard deviations (SDs) of three independent replicates. The data were analysed, and the Pearson correlation coefficient was calculated using SPSS Statistics 20.0 software. A value of *p* < 0.05 was considered to a significant difference. All the figures were generated by using Adobe Illustrator CS5 and Adobe Photoshop CC.

## 3. Results

### 3.1. Transcriptome Sequencing and De Novo Assembly

**Figure 1:**
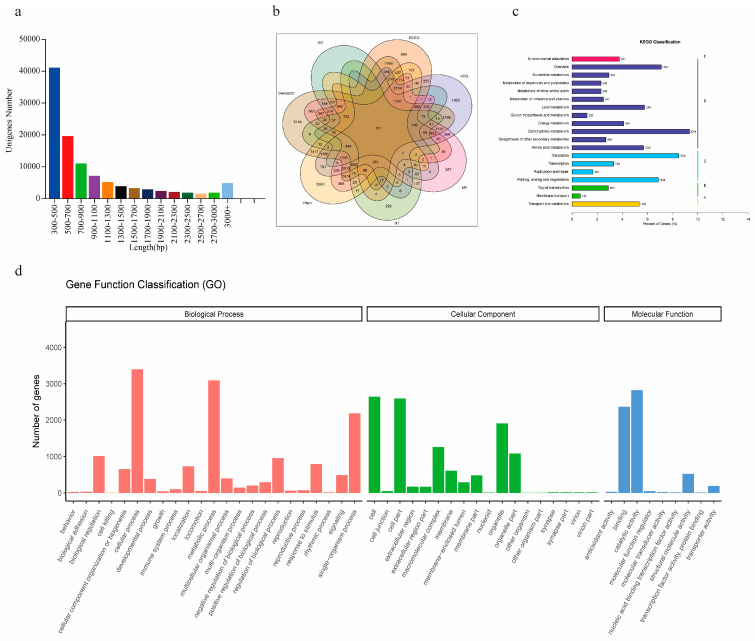
Summary of de novo assembly and functional annotation. (**a**) Length distribution of the unigenes. (**b**) Comparison numbers of annotated unigenes against different databases. (**c**) KEGG pathway classification of the unigenes. (**d**) GO annotation of all the unigenes.

To explore the gene expression variation and molecular mechanisms regulated by MeJA and identify the candidate genes involved in the SS biosynthetic pathway, transcriptome analysis was performed for *B. chinense* DC. with CK and MeJA treatment for different times (6 h, 12 h, 24 h, 48 h, and 72 h) using NovaSeq 6000 (Illumina, San Diego, CA, USA). After raw reads were quality filtered, a total of 932,271,610 clean reads of 18 libraries were obtained, and an overview of the high-quality reads for different libraries is shown in Table 1. In all the libraries, the clean read proportion was over 99.5%, the rRNA proportion was less than 0.5%, and the Q30 and Q20 values were above 89% and 95%, respectively. The average proportion of clean reads mapped to the reference transcriptome was 76.3%, indicating that the quality of the sequencing data met the standards for further analysis. A total of 104,057 unigenes with an average length of 965 bp were identified, and more than 60% of the unigenes were larger than 500 bp. The length distribution of the unigenes is shown in Figure 1a. The N50 length and GC content were 1384 bp and 38.23%, respectively.

### 3.2. Functional Annotation and Classification

For gene functional annotation, all the assembled unigenes were annotated by the GO, KEGG, KOG, NR, NT, Swiss-Prot, and Pfam databases with a cut-off e-value of 10^−5^. In total, 42,740 (41.07%) of the 104,057 unigenes were annotated, and the details of the annotation against each database are shown in Supplementary Table S2 and Figure 1b. The results showed that the SwissProt database annotated the highest percentages (79.02%) of unigenes, the NT database annotated the lowest percentage (7.09%) of unigenes, and 811 unigenes could be annotated by all the databases. Due to the lack of genomic data, 58.93% of the unigenes were not annotated by any of the seven databases.

Based on GO annotation, 6504 unigenes were classified as predicted functional genes of *B.chinense* DC. As shown in Figure 1d, these unigenes were classified and categorized into 50 functional groups belonging to the biological process (BP), cellular component (CC), and molecular function (MF) categories and were mostly enriched in the cellular process (52.30%), metabolic process (47.56%), catalytic activity (43.43%), and cell (40.65%) terms. In addition, the 12,187 genes annotated by KEGG analysis were grouped into five main categories, including cellular processes, environmental information processing, genetic information processing, metabolism, and organismal systems (Figure 1c). The metabolism category contained the most unigenes (82.52%), and the top four pathways were carbohydrate metabolism, translation, overview, folding, sorting and degradation. Some terpenoid and polyketide metabolism pathways related to the synthesis of SSs were also identified, including 503 unigenes.

### 3.3. Identification of DEGs

To explore the differences in *B. chinense* DC. at the transcriptome level in response to MeJA treatment, we identified DEGs between the CK and treatment groups. A total of 4053 DEGs were identified, and the pairwise comparative analysis of DEGs is shown in Supplementary Table S3. The results showed that the M12HR vs. CKR and M24HR vs. CKR comparison groups had 2527 (366 upregulated and 2621 downregulated) and 1736 (477 upregulated and 1289 downregulated) DEGs, respectively, significantly more than the other comparison groups. Moreover, most of the DEGs in 9 comparison groups were downregulated, most of the DEGs in 5 comparison groups were upregulated, and there were equal numbers of upregulated and downregulated DEGs between the M72HR and CKR groups.

**Figure 2:**
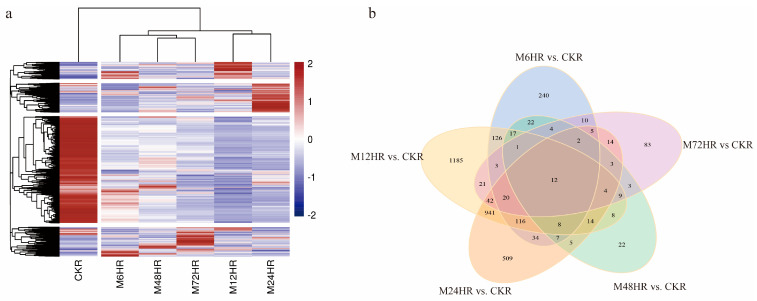
DEGs were identified in different comparisons. (**a**) Hierarchically clustered heatmap of DEG expression. (**b**) Venn diagrams of DEGs.

To visualize all the DEGs, the FPKM values of 4053 DEGs were used to generate a heatmap. As presented in Figure 2a, the samples treated with MeJA were separated from the control sample. All the DEGs were divided into four groups, and most of the DEGs were downregulated after MeJA treatment. A Venn diagram (Figure 2b) showed that there were 12 common DEGs in different comparisons between the control group and treated groups; most of them were upregulated and annotated as secondary metabolism-related signal transduction mechanisms, UDP-glucosyltransferase, and some proteins.

### 3.4. Enrichment Analysis of DEGs

**Figure 3:**
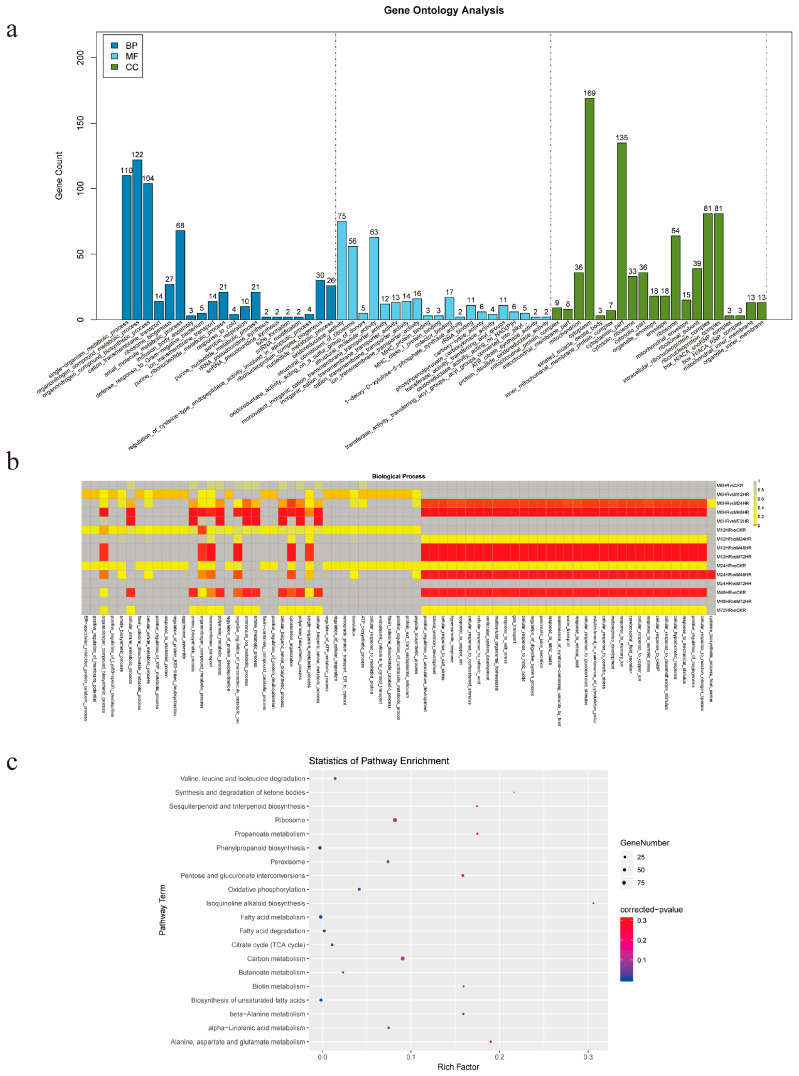
Enrichment analysis of DEGs. (**a**) GO enrichment analysis of DEGs. (**b**) GO enrichment analysis of BP terms. (**c**) KEGG enrichment analysis of the DEGs.

To understand the functions of all the DEGs, GO and KEGG pathway analyses were performed. The GO enrichment analysis showed that 89 BP terms, 68 CC terms, and 30 MF terms were significantly enriched, and the top 20 enriched terms are presented in Figure 3a. Because we focused on metabolism, the GO enrichment analysis of BP terms in each of the comparison groups was further visually depicted (Figure 3b), and 32 BP terms, which were mainly related to stress responses and development, were found to be significantly enriched in the 6 comparison groups.

Furthermore, all the DEGs were individually enriched in 116 KEGG pathways, and the 20 most significantly enriched KEGG pathways are shown in Figure 3c. Ribosome, carbon metabolism phenylpropanoid biosynthesis, fatty acid metabolism, and oxidative phosphorylation were the top five pathways with the largest numbers of enriched genes. In addition, other pathways involved in terpenoid metabolism were also enriched, including sesquiterpenoid and triterpenoid biosynthesis, terpenoid backbone biosynthesis, diterpenoid biosynthesis, monoterpenoid biosynthesis, ubiquinone and other terpenoid-quinone biosynthesis, indicating that the transcription of genes involved in terpenoid metabolism was responsive to MeJA treatment.

### 3.5. DEGs Involved in SS Biosynthetic Pathways

**Figure 4:**
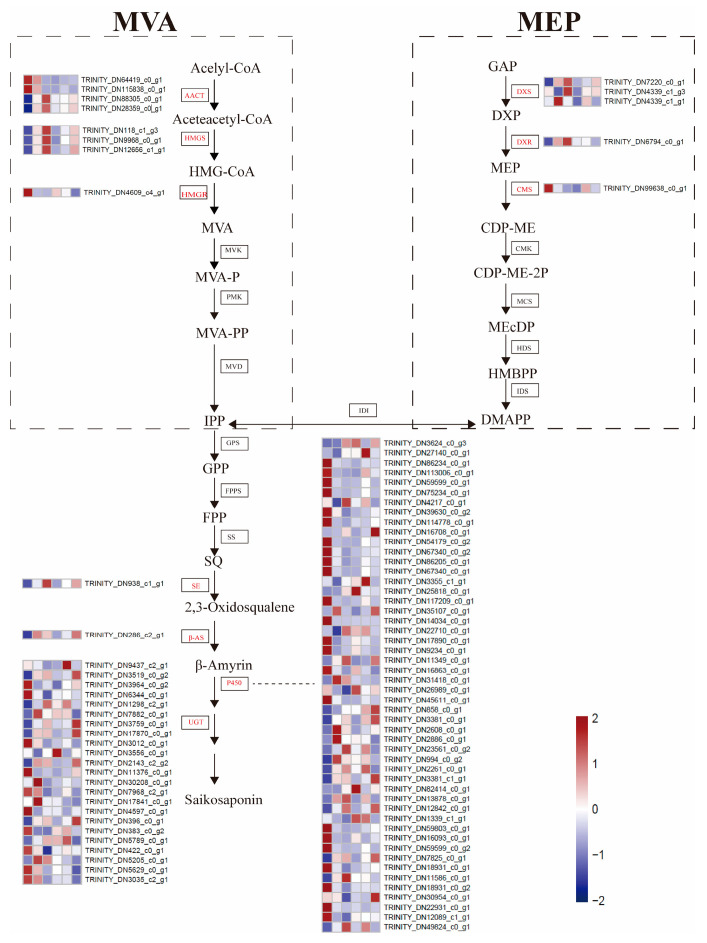
Transcript profiling of genes proposed to be involved in SS biosynthesis in *B. chinense* DC. The expression of individual genes was visualized as a heatmap, shown in the box; the red box indicates higher gene expression, and the blue box indicates lower gene expression. The samples are as follows from left to right: CKR, M6HR, M12HR, M24HR, M48HR, and M72HR.

SSs are a kind of natural terpenoid and are also the main biologically active components in *B. chinense* DC. Therefore, we further identified DEGs related to the synthesis of SSs to investigate the effect of exogenous MeJA on SS biosynthesis in *B. Chinense* DC. A total of 88 nonredundant DEGs were annotated to SS biosynthetic pathways, including 3 gene families in the MVA pathway, 3 gene families in the MEP pathway, and 4 gene families downstream of SS biosynthesis involved in skeleton construction and modifications (Figure 4). In the MVA and MEP pathways, 4 of the DEGs were encoded dacetoacetyl-CoA transferases (AACTs), 3 encoded HMG-CoA synthases (HMGSs), 1 encoded a HMG-CoA reductase (HMGR), 3 encoded 1-deoxy-d-xylulose-5-phosphate synthases (DXSs), 1 encoded a 1-deoxy-d-xylulose-5-phosphate reductoisomerase (DXR), and 1 encoded a 2-c-methyl-Derythritol 4-phosphate cytidylyl transferase (CMS). The pathways in the latter stages of SS biosynthesis had the highest number of DEGs identified, including 1 DEG encoding a squalene epoxidase (SE), 1 DEG encoding a β-AS, 52 DEGs encoding P450, and 24 DEGs encoding UGT.

Among these DEGs related to the synthesis of SSs, most DEGs in the MVA and MEP pathways showed significantly increased expression after MeJA treatment. In contrast, the expression of most DEGs downstream of SS biosynthesis were downregulated. Furthermore, a P450 gene (TRINITY_DN82414_c0_g1, log2FC = 4.39) showed an upregulated expression during MeJA treatment, with the highest upregulated expression levels observed in M24HR vs. CKR. Another P450 gene (TRINITY_DN14034_c0_g1, log2FC = −10.20) showed the highest downregulation in M12HR vs. CKR, also exhibiting sustained downregulation (log2FC = −10.20~−5.55) from 6 h to 72 h after MeJA treatment. In addition, these DEGs were further divided into five main clusters based on the normalized FPKM values (Supplementary Figure S2). Cluster 1 included 37 DEGs with sustained downregulation in all the treatment stages and belonged predominantly to the P450 and UGT gene families (89.2%).

### 3.6. Identification of TFs

**Figure 5:**
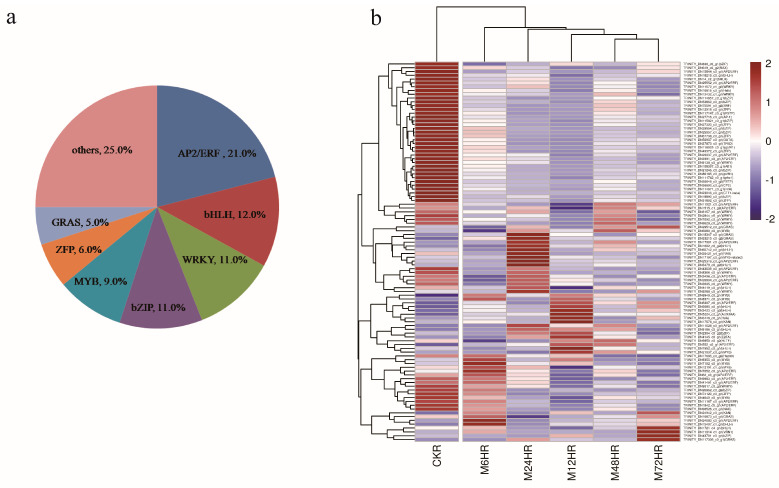
DEGs assigned to TFs. (**a**) Distribution of TF families. (**b**) Heatmap of DEGs encoding TFs.

TFs play an essential role in the regulation of gene expression and metabolic processes, and 1015 TFs belonging to 57 families were identified in this work. We also further analysed the DEGs encoding TFs. A total of 99 TF genes were identified, accounting for 2.4% of all the DEGs, and these were classified into 28 TF families according to the iTAK database. The top four families were AP2/ERF, bHLH, WRKY, and bZIP, with more than 10 genes for each family (Figure 5a). Hierarchical cluster analysis was also performed to further analyse these TFs (Figure 5b). The MeJA-treated groups clustered together, with different expression patterns in each treatment group. Approximately half of the TFs were downregulated after MeJA treatment, including AP2/ERF, WRKY, bZIP, ZFP, and bHLH. Moreover, the majority of the TFs showed treatment-time-specific expression patterns and could be divided into five distinct groups.

### 3.7. Integrated Analysis

**Figure 6:**
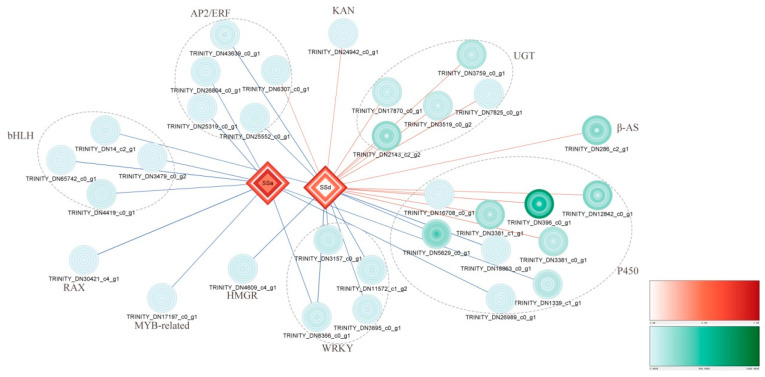
Integrated analysis of DEGs and SSs. The shapes represent different times of MeJA treatment and are as follows from inside to outside: CKR, M6HR, M12HR, M24HR, M48HR, and M72HR. The expression levels of DEGs (green, circle) and SSs (red, diamond) are represented by different shades of the colour. The red line represents a strongly positive correlation, blue represents a strongly negative correlation, and line thickness indicates the degree of correlation.

To further understand the regulatory mechanism of SSs at the different MeJA treatment stages, correlation analysis between 188 DEGs and SSs was performed, and 32 of the DEGs showed strong correlations with SSs (Supplementary Table S4 and Figure S3). A network map was built based on the Pearson correlation analysis, as shown in Figure 6. Most genes in the SS biosynthetic pathway exhibited a strong positive correlation and were downstream of the DEGs, which belonged to the UGT, β-AS, and P450 unigene families. Most of the TFs exhibited strong negative correlations, including the AP2/ERF, bHLH, RAX, MYB-related, and WRKY families. Among them, we found that a larger number of DEGs related to SSd, a P450 family DEG (TRINITY_DN3381_c0_g1), and a WRKY family DEG (TRINITY_DN8366_c0_g1) displayed the strongest positive and negative correlation with SSd, respectively. The WRKY family DEG (TRINITY_DN8366_c0_g1) also showed a negative correlation with SSa. Additionally, the changes in the expression levels of DEGs in the SS pathway were more significant than those of TFs at the different MeJA treatment stages. These DEGs that were strongly correlated with SSs were possibly important candidate genes that maybe associated with the biosynthesis and regulation of SSs.

### 3.8. Identification of SSRs and Variant Detection

To develop molecular markers of *B. chinense* DC., 104,057 assembled unigenes were used to mine potential microsatellites. A total of 41,956 SSRs of six types were detected, wherein dinucleotide repeats were the most abundant type, with 28,773 SSRs (Supplementary Figure S4), and 8950 sequences contained more than one SSR. Among the SSRs, AT/AT, AC/GT, A/T, and AG/CT were the top four nucleotide repeats, accounting for 57.8%, 31.9%, 29.3%, and 10.1% of the SSRs, respectively. The variants detected in this study included SNPs and InDels. A total of 783,814, 763,339, 764,452, 818,385, 789,266, and 817,775 SNPs were identified for CKR, M6HR, M12HR, M24HR, M48HR, and M72HR, respectively. For InDels, we identified 57,360 from CKR, 56,617 from M6HR, 57,838 from M12HR, 61,961 from M24HR, 59,043 from M48HR, and 61,393 from M48HR.

### 3.9. qRT–PCR Validation

**Figure 7:**
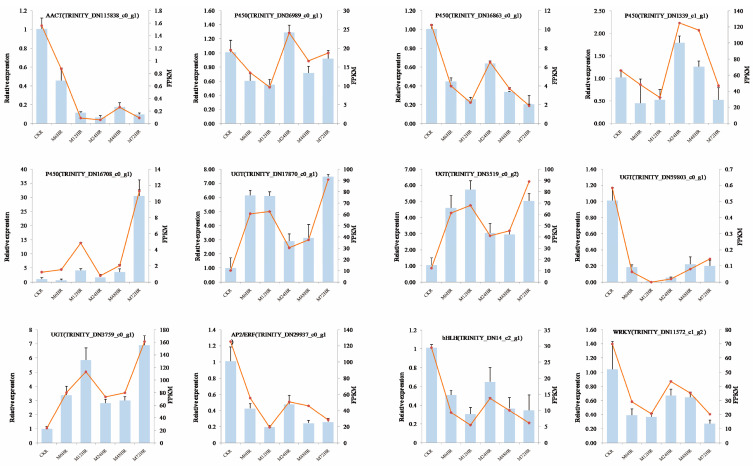
qRT–PCR validation of DEGs associated with SS biosynthesis and TFs. The blue columns represent relative expression determined with qRT–PCR (Y axis on the left), and the red lines represent the FPKM values of the transcriptome results (Y axis on the right).

To verify the reliability of the transcriptome data generated by RNA-seq, we selected 12 genes from the DEGs in the SS biosynthetic pathway and TFs for qRT–PCR assays. The qRT–PCR results of 12 genes supported our transcriptome data based on the FPKM results. Similar expression profiles were observed between the RNA-seq data and the qRT–PCR results (Figure 7), and the correlations between them were also high (average correlation coefficient = 0.97, Supplementary Table S5). These results indicated that the transcriptome data obtained by RNA-seq and the results of our analysis were reliable.

## 4. Discussion

Plant hormones play important roles in plant growth and development. MeJA is a commonly used plant hormone and is considered an important signalling molecule that can not only regulate the process of plant growth and development but also promote the biosynthesis of secondary metabolites, such as flavonoids, polysaccharides, and saponins [29,30,31,32]. In recent years, transcriptome technologies have been widely used to analyse the molecular mechanism of secondary metabolites under MeJA treatment, and many potential genetic pathways have been identified [8,33], providing the basis for further studies of the synthetic pathways associated with secondary metabolites. In this study, for the first time, we collected 18 samples of *B. chinense* DC. roots to comparatively analyse the transcript profiles under MeJA treatment at different times. In total, 104,057 reads were obtained, and 42,740 unigenes were successfully annotated. Among these unigenes, 4053 unigenes were identified as DEGs, including 88 DEGs involved in SS biosynthetic pathways (Figure 5) and 99 DEGs encoding TFs (Figure 6). The results showed that the numbers of DEGs among comparisons were different, 12 h and 24 h were the most notable time points (with 2527 and 1736 DEGs, respectively); this is likely due to the fact that *B. chinense* DC. requires change gene expressions at the early stage of MeJA treatment in order to adapt the MeJA stresses [34]. In plants, the DEGs associated with secondary metabolites can be further identified through correlation analyses between secondary metabolite concentrations and gene expression levels [35,36]. Hence, to gain further insight into the connection between DEG expression and the levels of SSs, we performed an integrated analysis by using the Pearson correlation coefficients (Figure 7). Approximately 32 DEGs showed strong correlations with SSs, indicating that they may be involved in SS accumulation in roots. Our study explored the effect of MeJA on SS synthesis, and we identified candidate genes related to SS biosynthesis in *B. chinense* DC., which will be helpful for further understanding the molecular mechanisms of SS biosynthesis.

It has been reported that the biosynthesis precursor of triterpenes occurs mainly via the MVA pathway in the cytoplasm but also depends on the MEP pathway in plastids [37]. In this study, we identified eight genes in three steps of the MVA pathway and five genes in three steps of the MEP pathway (Figure 5), and these genes were involved in the initial stages of the pathways. HMGS and DXR, which are important enzymes involved in the MVA and MEP terpenoid biosynthesis pathways, promote the production of terpenoids [38,39]. We found that three HMGSs and one DXR were significantly upregulated in the initial stage after MeJA treatment, which was consistent with previous research [39,40], indicating that the precursor supply was active under MeJA treatment. Notably, there was an inconsistency in that expression levels of HMGS and DXR peaked at different times, and this discrepancy may have been due to species-related differences. β-AS is supposed to be the branching enzyme in the biosynthesis of triterpene saponins and is responsible for catalysing the conversion of 2,3-oxidosqualene to the triterpenoid skeleton β-amyrin [41]. Only one β-AS (TRINITY_DN286_c2_g1) was identified in our study, and the expression of β-AS was also significantly positively correlated with the contents of SSs in the correlation analysis. Interestingly, β-AS (TRINITY_DN286_c2_g1) showed a high similarity of approximately 95.35% to another β-AS (Bc61215), which was previously identified and has been functionally characterized in *Pichia pastoris* as a monofunctional β-AS [42], suggesting that the two β-ASs may actually represent the same gene. The number of DEGs involved in the downstream pathway for SS biosynthesis was the largest, with a total of 76 DEGs, including the P450 and UGT families. Similar results were also observed in other studies [43]. Moreover, nine P450s and five UGTs were significantly correlated with the SS contents, and these genes may be important at the late stage of modification in SS biosynthesis in roots. Our results suggested that MeJA regulated SS biosynthesis at different stages and that the regulation of MeJA occurred via a complex regulatory network [44]. Therefore, further study needs to be conducted on the influence of other metabolic aspects on SSs.

TFs play important roles in plant growth and development and are also considered regulators of gene expression. Previous works have revealed that saponin biosynthesis is regulated by TFs [45,46]. Several TFs were predicted in the genus *Bupleurum*, and the top families included C3H, AP2-EREBP, MYB-related, bHLH, NAC, MYB, and bZIP [13,16]. In *B. chinense*, the levels of SSs were decreased by overexpression of BcbZIP134 in hairy roots [24], and the expression of four WRKY TFs showed a consistent trend in terms of SS contents after treatment with NaCl and PEG6000 [47]. Our results showed that 99 TF genes, including AP2/ERF, bHLH, WRKY, RAX, and bZIP, were identified as DEGs induced by MeJA, and 13 of them showed a highly significant correlation with the SS contents. Furthermore, correlation analysis showed a significant negative correlation between the contents of SSs and the WRKY family, and the same trend was also found for four bHLH TFs. These WRKY and bHLH TFs might negatively regulate SS synthesis, which needs to be further verified in the future.

## 5. Conclusions

In summary, comparative transcriptome analysis was performed to investigate the molecular mechanisms corresponding to the response of *B. chinense* DC. roots to MeJA. A total of 104,057 unigenes were obtained, and 42,740 unigenes were annotated. The DEGs among the pairwise comparisons were identified and functionally analysed, and the results indicated that most of these genes were related to stress responses and development. In particular, we compared DEGs involved in SS biosynthesis and TFs. Approximately 88 DEGs encoding 10 key enzymes involved in the SS biosynthetic pathway were identified. Approximately 99 DEGs were identified as TF genes, most of which were downregulated after MeJA treatment. Furthermore, 32 DEGs showed strong correlations with SSs, which may be involved in the regulation of SS biosynthesis. These findings may further expand the number of possible candidate genes in the SS biosynthesis pathway and contribute to a better understanding of the effect of MeJA on *B. chinense* DC. The results could provide a crucial basis for genetic engineering, metabolic engineering, and molecular breeding in plants of the *Bupleurum* genus.

## Figures and Tables

**Table 1:** Summary of Illumina RNA-sequencing data.

Sample Name	Clean Reads	Clean Bases (bp)	Clean Reads (%)	Clean GC (%)	Clean Q20 (%)	Clean Q30 (%)	rRNA Ratio (%)	Mapped Ratio (%)
CKR1	44,526,918	6,679,037,700	99.65	43.01	97.20	92.51	0.35	75.9
CKR2	42,887,626	6,433,143,900	99.71	42.87	97.22	92.72	0.29	75.83
CKR3	53,660,932	8,049,139,800	99.75	42.83	97.26	92.94	0.25	74.66
M6HR1	42,239,902	6,335,985,300	99.67	42.88	96.87	91.99	0.33	76.9
M6HR2	54,916,582	8,237,487,300	99.66	43.12	97.05	92.11	0.34	76.02
M6HR3	57,836,632	8,675,494,800	99.68	43.03	97.04	92.26	0.32	76.41
M12HR1	45,061,582	6,759,237,300	99.58	42.78	96.89	92.07	0.42	76.51
M12HR2	54,093,938	8,114,090,700	99.54	42.92	97.19	92.58	0.46	76.85
M12HR3	56,753,582	8,513,037,300	99.55	42.88	96.82	91.87	0.45	76.78
M24HR1	50,885,024	7,632,753,600	99.57	42.84	97.37	93.13	0.43	76.06
M24HR2	54,550,916	8,182,637,400	99.68	42.85	96.73	91.70	0.32	76.53
M24HR3	65,380,004	9,807,000,600	99.60	42.72	97.29	92.95	0.40	75.59
M48HR1	45,675,820	6,851,373,000	99.69	42.93	97.24	92.84	0.31	77.19
M48HR2	51,196,014	7,679,402,100	99.64	42.96	97.23	92.83	0.36	75.92
M48HR3	60,980,926	9,147,138,900	99.52	42.96	97.34	92.96	0.48	76.38
M72HR1	48,107,866	7,216,179,900	99.50	43.08	95.98	89.99	0.50	75.8
M72HR2	48,412,486	7,261,872,900	99.65	42.79	97.33	92.76	0.35	76.52
M72HR3	55,104,860	8,265,729,000	99.55	43.05	97.15	92.69	0.45	77.62

## Data Availability

Not applicable.

## Supplementary Materials

The following Supporting Information can be downloaded at: https://www.mdpi.com/article/10.3390/life13020563/s1, Table S1: Functional annotation of assembled unigenes; Table S2: Statistical analysis of DEGs between groups; Table S3: Pearson correlation with SSs; Table S4: The correlations of qRT–PCR results and transcriptome sequencing data; Table S5: List primers sequence used for qRT–PCR. Figure S1: The structure of SSa and SSd; Figure S2: Hierarchical cluster analysis of all DEGs involved in SSs biosynthesis; Figure S3: SSa and SSd content in roots of *Bupleurum chinense* DC.; Figure S4: Distribution of SSR motifs in different types.
