# Supplementary material for: Comparative Transcriptome Analysis Provides Insights into the Molecular Mechanism Underlying the Effect of MeJA Treatment on the Biosynthesis of Saikosaponins in Bupleurum chinense DC
Source: Life (Basel). 2023 Feb 17;13(2):563. doi: 10.3390/life13020563 (PMC9960380; doi:10.3390/life13020563)
Supplement: Supplementary file 1 [file life-13-00563-s001.zip › Table S1.pdf]

Table.S1 Functional annotation of assembled unigenes.

Database	Number	Percentage (%)	0<=length<1000	1000<=length<2000	2000<=length<3000	3000<=length<6000	length>=6000
GO	6504	15.22	3290	1576	941	653	44
KEGG	12187	28.51	6705	2998	1456	947	81
KOG	22138	51.80	11380	5458	3067	2034	199
NR	23493	54.97	9576	6382	4170	3067	298
NT	3029	7.09	1819	606	306	257	41
Swissprot	33773	79.02	15931	8855	5192	3479	316
Pfam	27319	63.92	10433	8075	5084	3419	308
All	42740	100.00	21545	10955	6020	3883	337
