# Supplementary material for: Comparative Transcriptome Analysis Provides Insights into the Molecular Mechanism Underlying the Effect of MeJA Treatment on the Biosynthesis of Saikosaponins in Bupleurum chinense DC
Source: Life (Basel). 2023 Feb 17;13(2):563. doi: 10.3390/life13020563 (PMC9960380; doi:10.3390/life13020563)
Supplement: Supplementary file 1 [file life-13-00563-s001.zip › Table S2.pdf]

Table.S2 Statistical analysis of DEGs between groups.

Group comparison	DEGs number		
	**All**	**Up**	**Down**
M6HR vs CKR	627	235	392
M6HR vs M12HR	322	208	114
M6HR vs M24HR	596	204	392
M6HR vs M48HR	111	57	54
M6HR vs M72HR	137	79	58
M12HR vs CKR	2527	366	2161
M12HR vs M24HR	301	97	204
M12HR vs M48HR	123	44	79
M12HR vs M72HR	95	42	53
M24HR vs CKR	1736	447	1289
M24HR vs M48HR	132	85	47
M24HR vs M72HR	108	68	40
M48HR vs CKR	141	59	82
M48HR vs M72HR	34	11	23
M72HR vs CKR	236	118	118
