# Supplementary material for: Comparative Transcriptome Analysis Provides Insights into the Molecular Mechanism Underlying the Effect of MeJA Treatment on the Biosynthesis of Saikosaponins in Bupleurum chinense DC
Source: Life (Basel). 2023 Feb 17;13(2):563. doi: 10.3390/life13020563 (PMC9960380; doi:10.3390/life13020563)
Supplement: Supplementary file 1 [file life-13-00563-s001.zip › Table S3.pdf]

Table.S3 Pearson correlation with SSs.

Var1	Var2	family	cor
SSd	TRINITY_DN43639_c0_g1	AP2/ERF	-0.87657
SSa	TRINITY_DN25319_c0_g	AP2/ERF	-0.8712
SSd	TRINITY_DN6307_c0_g1	AP2/ERF	0.848262
SSd	TRINITY_DN25552_c0_g1	AP2/ERF	-0.82594
SSa	TRINITY_DN26804_c0_g1	AP2/ERF	-0.82326
SSd	TRINITY_DN286_c2_g1	β-AS	0.88605
SSa	TRINITY_DN65742_c0_g1	bHLH	-0.89978
SSa	TRINITY_DN3479_c0_g2	bHLH	-0.85667
SSa	TRINITY_DN4419_c0_g1	bHLH	-0.85026
SSd	TRINITY_DN14_c2_g1	bHLH	-0.81485
SSd	TRINITY_DN4609_c4_g1	HMGR	-0.89442
SSd	TRINITY_DN24942_c0_g1	KAN	0.857035
SSa	TRINITY_DN17197_c0_g1	MYB related	-0.84104
SSd	TRINITY_DN3381_c0_g1	P450	0.982778
SSd	TRINITY_DN396_c0_g1	P450	0.966217
SSd	TRINITY_DN3381_c1_g1	P450	0.946268
SSd	TRINITY_DN16863_c0_g1	P450	-0.91033
SSd	TRINITY_DN12842_c0_g1	P450	0.905297
SSa	TRINITY_DN26989_c0_g1	P450	-0.86321
SSd	TRINITY_DN5629_c0_g1	P450	-0.83115
SSd	TRINITY_DN16708_c0_g1	P450	0.822907
SSa	TRINITY_DN1339_c1_g1	P450	-0.81341
SSa	TRINITY_DN30421_c4_g1	RAX	-0.95719
SSd	TRINITY_DN7825_c0_g1	UGT	0.964072
SSd	TRINITY_DN17870_c0_g1	UGT	0.925463
SSd	TRINITY_DN3519_c0_g2	UGT	0.917424
SSd	TRINITY_DN3759_c0_g1	UGT	0.907372
SSd	TRINITY_DN2143_c2_g2	UGT	0.866755
SSd	TRINITY_DN8366_c0_g1	WRKY	-0.92263
SSd	TRINITY_DN11572_c1_g2	WRKY	-0.87065
SSa	TRINITY_DN8366_c0_g1	WRKY	-0.84875
SSd	TRINITY_DN3157_c0_g1	WRKY	-0.83962
SSd	TRINITY_DN3695_c0_g1	WRKY	-0.83299
