# Supplementary material for: Comparative Transcriptome Analysis Provides Insights into the Molecular Mechanism Underlying the Effect of MeJA Treatment on the Biosynthesis of Saikosaponins in Bupleurum chinense DC
Source: Life (Basel). 2023 Feb 17;13(2):563. doi: 10.3390/life13020563 (PMC9960380; doi:10.3390/life13020563)
Supplement: Supplementary file 1 [file life-13-00563-s001.zip › Table S4.pdf]

Table.S4 The correlations of qRT-PCR results and transcriptome sequencing data.

GeneID	correlation
TRINITY_DN115838_c0_g1	0.99
TRINITY_DN17870_c0_g1	0.98
TRINITY_DN16863_c0_g1	0.99
TRINITY_DN1339_c1_g1	0.94
TRINITY_DN26989_c0_g1	0.96
TRINITY_DN16708_c0_g1	0.96
TRINITY_DN3519_c0_g2	0.92
TRINITY_DN59803_c0_g1	0.99
TRINITY_DN3759_c0_g1	0.97
TRINITY_DN29937_c0_g1	0.97
TRINITY_DN14_c2_g1	0.97
TRINITY_DN11572_c1_g2	0.98
