# Supplementary material for: Comparative Transcriptome Analysis Provides Insights into the Molecular Mechanism Underlying the Effect of MeJA Treatment on the Biosynthesis of Saikosaponins in Bupleurum chinense DC
Source: Life (Basel). 2023 Feb 17;13(2):563. doi: 10.3390/life13020563 (PMC9960380; doi:10.3390/life13020563)
Supplement: Supplementary file 1 [file life-13-00563-s001.zip › Table S5.pdf]

Table S5. List primers sequence used for qRT-PCR.

Primer code	Primer Sequence (5'→3')
RT115838-F	AAAGAGCAAGACGACTACGC
RT115838-R	AACAATGTCGCCCTTCTTCG
RT26989-F	AAACCAACCACCTTCACCAC
RT26989-R	ACAGGCACAGTTCCAAAGTG
RT16863-F	AGCCCATGTTTGGTAATGGC
RT16863-R	ACCATTAAGCTTCCCATGGC
RT1339-F	AGCAAGTTAGACACGTTGGC
RT1339-R	TGTTGTGAAGGCCAGCTTTC
RT16708-F	TGGCCACTTGGAGAGTCATTAC
RT16708-R	AGCTTTACCAGTAGGGCCTTTC
RT-17870-F	TGTGCCTGTGCTTGTTATGC
RT-17870-R	TCACACACTTTGCGACTTCC
RT-3519-F	AATGGGCAATCAAGGTGCTC
RT-3519-R	AAGCGTTGAGAGGAGTTGTG
RT59803-F	ATGTAATGCCCACACAACGG
RT59803-R	TCTGCGGTTTTAGGCAATGC
RT3759-F	AACGCGCCATGCAAGATTTC
RT3759-R	AAGCGAGGGATGTTTAGAGTGG
RT29937-F	TCAAGCGGCGTATTCAATGC
RT29937-R	ACACCCTGCCTCACAACTATG
RT14-F	TTGGCACCTTCTGCTCAAAC
RT14-R	AGAATGGCTTGTGTCATCGC
RT11572-F	GCAATCACACAGTGGAGTGAAC
RT11572-R	ACCTAGGAAAAGTGGCTCCAAG
RTADF5-F	CGAGTTCCCTCTTGAATCTGTC
RTADF5-R	TGAGGATTTCACTGCCTGTCTA
