# Supplementary figures and images for: Comparative Transcriptome Analysis Provides Insights into the Molecular Mechanism Underlying the Effect of MeJA Treatment on the Biosynthesis of Saikosaponins in Bupleurum chinense DC
Source: Life (Basel). 2023 Feb 17;13(2):563. doi: 10.3390/life13020563 (PMC9960380; doi:10.3390/life13020563)

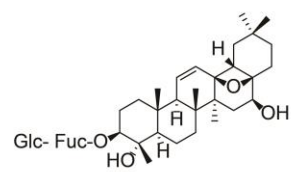

saikosaponin a

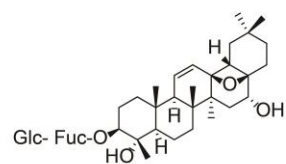

saikosaponin d

**Figure S1.** The structure of SSa and SSd.

Supplement: Supplementary file 1 [file life-13-00563-s001.zip › Figure S1.pdf]

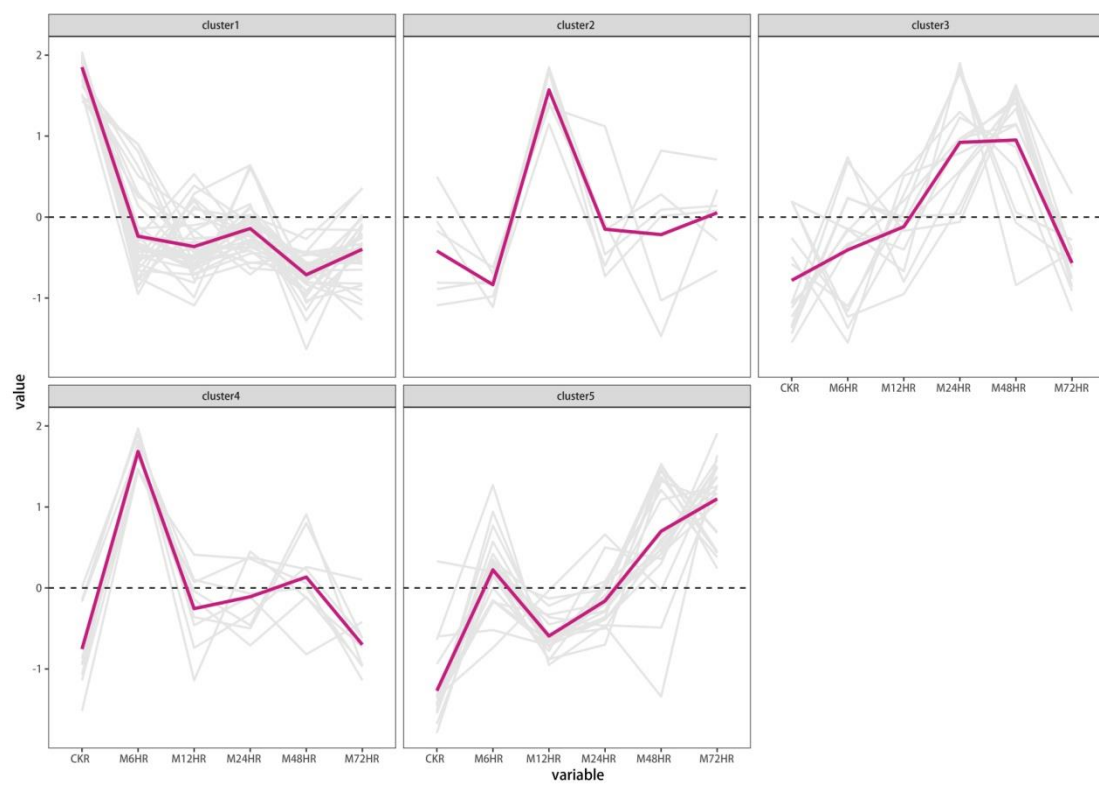

**Figure S2.** Hierarchical cluster analysis of all DEGs involved in SSs biosynthesis.

Supplement: Supplementary file 1 [file life-13-00563-s001.zip › Figure S2.pdf]

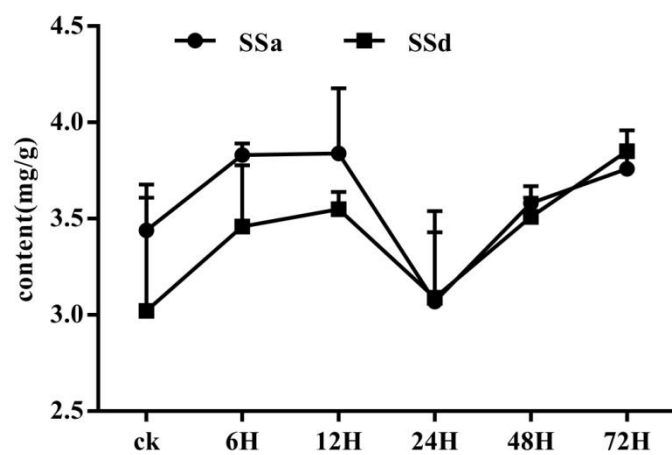

**Figure S3.** SSa and SSd content in roots of *Bupleurum chinense* DC..

Supplement: Supplementary file 1 [file life-13-00563-s001.zip › Figure S3.pdf]

## Distribution of SSR Motifs

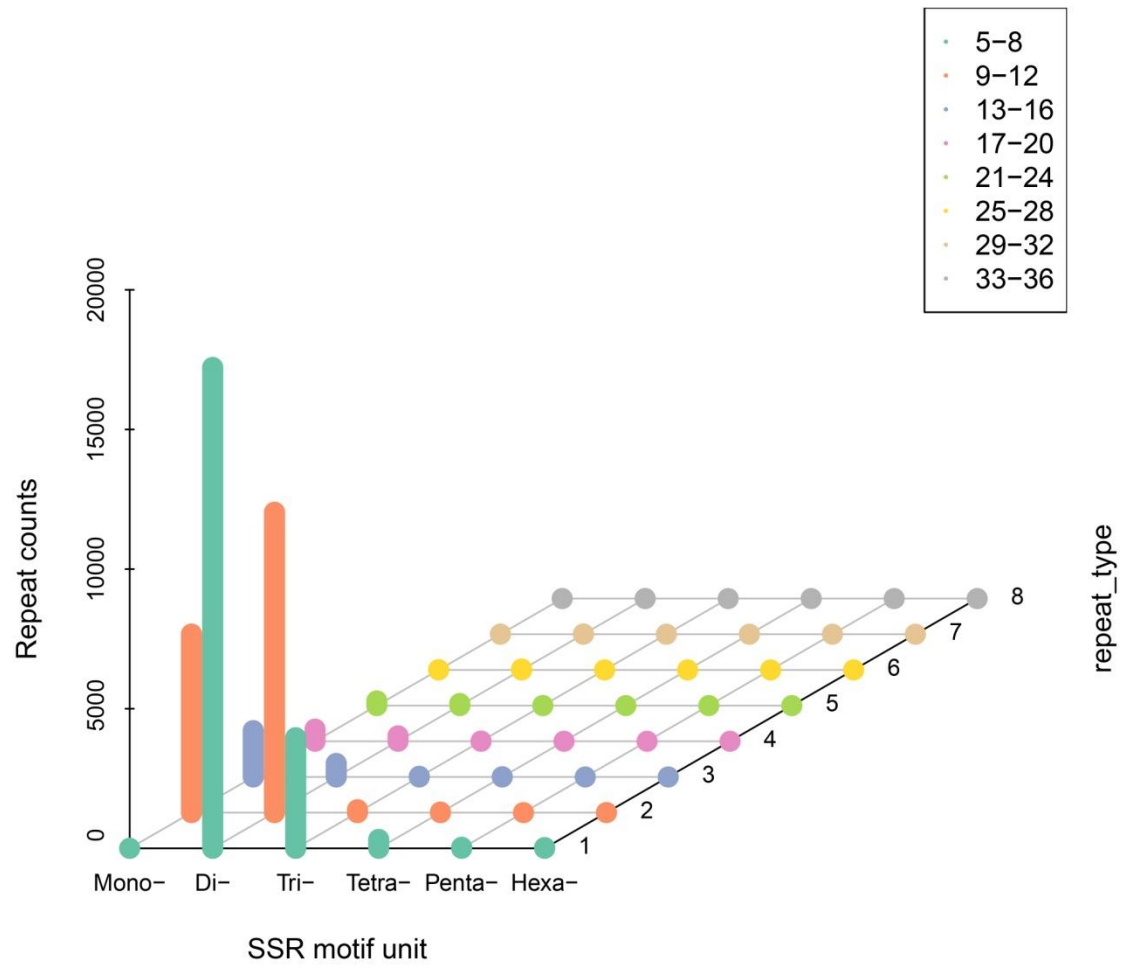

**Figure S4.** Distribution of SSR motifs in different types.

Supplement: Supplementary file 1 [file life-13-00563-s001.zip › Figure S4.pdf]
